# Effects of Feeding Regimes and Postmortem Aging on Meat Quality, Fatty Acid Composition, and Volatile Flavor of Longissimus Thoracis Muscle in Sunit Sheep

**DOI:** 10.3390/ani12223081

**Published:** 2022-11-09

**Authors:** Zhihao Yang, Chang Liu, Lu Dou, Xiaoyu Chen, Lihua Zhao, Lin Su, Ye Jin

**Affiliations:** College of Food Science and Engineering, Inner Mongolia Agricultural University, Hohhot 010018, China

**Keywords:** sheep, feeding regimes, postmortem aging, oxidative stability, meat quality, fatty acid, volatile flavor compounds

## Abstract

**Simple Summary:**

This manuscript investigated the effects of different feeding regimes on antioxidant activity, meat quality, fatty acid composition, lipid oxidation, and volatile matter production in the longissimus thoracis muscle of Sunit sheep during postmortem aging. It was found that pasture-fed sheep had better oxidative stability, meat quality, and fatty acid composition. At the same time, aging affected the oxidative stability, meat quality, and fatty acid composition of sheep meat, which in turn had an impact on the flavor composition of sheep meat. Aging also eliminates the differences caused by different feeding regimes to a certain extent. The purpose of this study was to investigate the effects of feeding regimes and aging time on the meat quality of sheep and to provide meat professionals with a theoretical basis that can be applied in practical production. It also accelerated the transition from traditional grazing to indoor rearing to maintain ecological balance.

**Abstract:**

The effects of different feeding regimes on antioxidant activity, meat quality, fatty acid composition, lipid oxidation, and volatile matter production in the longissimus thoracis (LT) of Sunit sheep at 0, 24, 48, 72, and 96 h postmortem were investigated. The results showed that the activity of antioxidant enzymes, tenderness, water retention, and percentage of unsaturated fatty acids were significantly higher in the pasture-fed sheep (PF) than in the concentrate-fed sheep (CF) (*p* < 0.05). During postmortem aging, antioxidant activity, water retention, and the proportion of unsaturated fatty acids decreased in the PF and CF (*p* < 0.05), while malondialdehyde (MDA) content, the proportion of saturated fatty acids, and the content of flavor substances resulting from fat oxidation increased. After 24 h of LT muscle aging, the pH and shear force of the meat started to increase and the color stabilized. The differences between shear force values and lipid volatile flavor substance content of sheep meat under different feeding regimes disappeared with increasing aging time. PF had better oxidative stability and fatty acid composition. Postmortem aging changed the oxidative stability of sheep meat, thus affecting meat quality and fatty acid composition and consequently meat flavor composition, while aging also eliminated to some extent the differences caused by feeding regimes.

## 1. Introduction

Sunit sheep is an excellent group formed by natural selection and artificial breeding in the specific growth environment of the Sunit steppe. It belongs to Mongolian sheep and has good meat production performance, nutritional quality, and edible quality [1,2,3]. In China, sheep-breeding areas are mainly distributed in the western and northwestern regions, where the ecosystem is fragile. In response to the degradation of the natural environment, the state has implemented a policy of returning grazing areas to grassland, which has transformed sheep farming in many areas from a traditional grazing mode to an intensive mode [4,5].

The change of feeding regimes have a great influence on the meat quality of sheep [6,7]. Some studies have found that meat from sheep raised on pasture feeding is considered to be better in terms of its polyunsaturated fatty acid (PUFA) profile and maintenance of oxidation stability [8,9]. Wang’s study showed that pasture feeding could influence the composition of short-chain fatty acids in lambs by regulating the composition of rumen bacterial community in lambs [10]. In addition, the study found that grass-fed sheep had better meat quality than concentrate-fed sheep: the latter had higher levels of subcutaneous and intramuscular fat [11]. Studies on flavor fingerprinting mainly focus on the effects of feeding regimes on volatile compounds [12,13] and specific plant-derived compounds, namely terpenoids, plant alcohols, and indoles [14,15]. However, little work has been done to analyze lipid flavor precursors and volatile compounds in detail. Therefore, revealing the flavor properties from the perspective of precursors and volatile compounds may be more helpful to understand the effect of the feeding regime on flavor.

In addition to feeding regimes, postmortem aging time significantly affects product quality [16,17,18]. In a low-temperature environment, the aging process causes the meat surface to form a dry oil film, which can reduce the loss of water in the muscle and make the meat soft and juicy. At the same time, it prevents the invasion and reproduction of microorganisms and extends the storage period of meat. [19]. In addition, the meat itself contains certain flavor precursor compounds, which undergo a series of chemical changes during ripening, which can significantly improve the flavor and overall acceptance of meat [20]. However, the fat oxidation caused by the aging time of the process may have adverse effects on the nutrition and flavor characteristics of meat [21]. Therefore, determining the appropriate aging time is very important to the quality, fatty acid composition, and volatile flavor components of sheep meat.

The purpose of this study was to determine the effects of two feeding regimes on meat quality, antioxidant capacity, fatty acid profile, and volatile flavor composition of Sunit sheep. At the same time, the meat quality, oxidation state, fatty acid spectrum, and volatile compound composition of sheep meat were evaluated after aging for 24, 48, 72, and 96 h to clarify the influence of aging time on various indexes of sheep meat, thus providing a theoretical reference and technical support for improving the quality of sheep meat.

## 2. Materials and Methods

### 2.1. Animals, Experimental Design and Diets

This study was approved by the Experimental Animal Welfare and Ethics Committee of Inner Mongolia Agricultural University and complied with the National Research Council’s Guide for the Care and Use of Laboratory Animals (approval document number IMAU2021072). Briefly, 24 Sunit sheep were randomly selected (90 days old; all castrated rams; initial weight: 18.79 ± 1.54 kg). The experimental sheep were randomly assigned to the pasture-fed sheep (PF) and concentrate-fed sheep (CF) groups. PF: sheep grazed on 5.33 km^2^ of semi-desert grassland (*Stipa gobicao*, *Stipa breviflora*, and *Cleistogenes squarrosa*) for 12 h per day (8: 00 a.m. and 8: 00 p.m.), with freedom to move around and fed on fresh grass. The amount of exercise is 12.32 ± 0.65 km. CF: 12 sheep per pen, 6 × 7 m per pen, 3.5 m^2^ per sheep; fed twice a day (8: 00 a.m. and 8: 00 p.m.) on concentrated feed with consisting of corn stalks, skin of sunflower seed, and adding corn’s concentrate and fattening feed, with freedom to move around. The amount of exercise was 2.53 ± 0.35 km. The diet composition of the PF and CF group is shown in Table 1. The formal trial lasted for 90 days after 7 days of adaptive diet. Water was provided ad libitum throughout the experiment.

### 2.2. Sample Collection

At the end of the experiment, the selected lambs (weight at slaughter: PF: 31.63 ± 1.82 kg; CF: 32.51 ± 1.56 kg) were fasted for 12 h and then transported to a commercial slaughterhouse for slaughtering. After each sheep was peeled, the *longissimus thoracis (LT)* muscle on the left side of the carcass was taken from the 15 min and cut into small pieces. One sample was taken from each carcass as a sample for 0 h. The rest of the muscle pieces were aged without packaging and were directly exposed to air at a temperature of 2~4 °C, wind speed 0.25~0.5 m/s, and humidity of 85~90% for 24, 48, 72, and 96 h. The pH, shear force (N), color, cooking loss (%), fatty acid composition, and volatile flavor compounds of the samples were determined at each sampling point (0, 24, 48, 72, and 96 h after slaughter). At the same time, about 5 g samples were taken and placed in a sterile enzyme-free tube for storage in liquid nitrogen and then transferred to the refrigerator at –80 °C in the laboratory for the determination of the degree of lipid oxidation and the activity of antioxidant enzymes. All determinations were repeated in triplicate.

### 2.3. Evaluation of Antioxidant Activity

Muscle samples (0.5 g) taken at 0, 24, 48, 72, and 96 h after postmortem were weighed, added to 4.5 mL 0.85% saline solution, and then homogenized three times (55 Hz) for 30 s each (Ystral homogenizer, Series X10/25, Ystral, Ballrechten-Dottingen, Germany). After the samples were homogenized well, they were collected by centrifugation (Model CPR, Beckman Coulter, Brea, CA, USA) at 3000 rpm/min for 10 min at 4 °C, and the supernatants were collected for analytical determination. Enzyme analyses were conducted for the meat samples using kits from Nanjing Jiancheng (Nanjing, Jiangsu, China) according to the methodology proposed in each kit’s extraction protocol.

A total antioxidant capacity assay kit (T-AOC) was used for analysis (colorimetric method: No. A015).

Glutathione peroxidase (GSH-PX) was measured using a GSH-PX assay kit (colorimetric method: No. A005-1).

The superoxide dismutase (SOD) activity was measured using an SOD assay kit (WST-1 method: No. A001-3).

Catalase (CAT) activity was measured using a CAT assay kit (colorimetric method: No. A007-2-1).

### 2.4. Meat Quality Measurement

The pH of muscle was measured at 0, 24, 48, 72, and 96 h postmortem using a portable pH meter (pH-Star; Ingenieurbüro R. Matthäus, Ebenried, Germany), which was calibrated in buffer solutions with pH of 4.60 and 7.00 and inserted into the center of each meat sample. Each sample was measured three or more times.

Shear force: Small muscle samples (approximately 6 cm × 2 cm × 2 cm) were removed from samples 0, 24, 48, 72, and 96 h and individually placed inside polyethylene bags and then cooked in a 75 °C water bath until a core temperature of 70 °C was reached, and samples were then kept for 20 min. During cooking, the core temperature of samples was tracked using a digital thermometer (DM6801A, Shenzhen Victor Hi-tech Co. Ltd., China). About 6 to 8 1.27 cm diameter cylindrical cores parallel to the muscle fiber orientation were removed from each muscle sample. A single, peak shear force measurement was obtained for each core using a Warner-Bratzler meat shear machine (C-LM3B; Northeast Agricultural Univ., Harbin, China), and an average shear force was calculated and recorded for each muscle sample. The results were expressed as load in Newton (N).

Cooking loss (%): At 0, 24, 48, 72, and 96 h of aging, the samples were cooked individually in plastic bags immersed in a water bath at 75 °C until they reached an internal temperature of 70 °C and then held at for 20 min. During cooking, the core temperature of samples was tracked using a digital thermometer (DM6801A, Shenzhen Victor Hi-tech Co. Ltd., Shenzhen, China). Then, cooking loss was calculated as the percentage weight loss before and after heating.

The color of fresh and aged samples was measured on the meat surface. The color of samples was determined using a colorimeter (Chroma meter, CR-410, Tokyo, Japan) equipped with a standard D65 light source, using a standard observer’s 2° position with a pulsed xenon lamp and an 8 mm reading surface area. The lightness (L*), redness (a*), and yellowness (b*) for each sample were recorded three times, and the average value was calculated.

### 2.5. Fatty Acid Analysis

Lipids were extracted according to the method described in Folch, Lees, and Stanley [22]. The LT muscle sample was thawed. A 5 g sample was placed in a triangular flask, and an extraction solvent (2:1 chloroform/methanol, *v*/*v*) was added. The sample was heated and stirred for 2 h and then passed through a G3 funnel overnight. The filtrate was thoroughly mixed with 5 mL 2% NaCl solution, and after standing for stratification, the bottom liquid was removed. An appropriate amount of Na_2_SO_4_ solution was added, and the solution was concentrated and evaporated to obtain crude fat. Then, fat saponification was performed (the crude fat was added to 5 mL 2% CH_3_OH-NaOH solution, which was then placed in a 70 °C water bath for 5 min) followed by methyl esterification (3 mL BF_3_-C_4_H_10_O solution was added to the sample after saponification, which then placed in a 70 °C water bath for 2 min). Then, the samples were mixed with 2 mL n-hexane solution, placed in a 70 °C water bath for 1 min, and the 5 mL saturated NaCl was then added in a dropwise manner. The solution was allowed to stand for 10 min, and then, an appropriate amount of Na_2_SO_4_ solution was added. Finally, the resulting solution was filtered through an organic filter membrane and placed in a sample bottle for later measurement.

The quantification of fatty acid methyl esters (FAMEs) was conducted on a gas chromatography system (Scion 456-GC, Bruker, Billerica, MA, USA) equipped with a flame ionization detector and an SP-2560 fused silica capillary column (100 m × 0.25 mm, 0.20 µm). The FAMEs were identified using a standard mixture containing 37 FAMEs.

### 2.6. Determination of Lipid Peroxidation

The lipid peroxidation products of thiobarbituric acid reactive substances in meat sample extracts were evaluated according to the method described by Uchiyama [23]. Thiobarbituric acid reactive substances were expressed as a malondialdehyde (MDA) equivalent, and the absorbance was measured at a wavelength of 532 nm using a microplate reader (infinite M200, Tecan, Switzerland). An MDA content determination kit was used in the analysis (TBA method: A003-1). The kit was obtained from the Nanjing Jiancheng Institute of Bioengineering (Nanjing, Jiangsu, China), and values were expressed as mg (MDA)/kg.

### 2.7. Volatile Organic Compounds

Volatile flavor compounds were determined using the method established by Vasta et al. [24]. The sample was thawed in the natural state, the connective tissue removed and cut into diced meat and then chopped into minced meat, and accurately weighed 5 g in a 20 mL sample bottle; the bottle cap was tightened, and the sample was placed in a water bath at 60 °C, inserted into the aging extraction head, adsorbed for 40 min, and then directly inserted into the injection port of GC-MS, desorbed at 250 °C for 3 min.

The gas chromatographic conditions were as follows: DB-5 column (30 m × 0.25 mm, 0.25 μm). The carrier gas was helium at a flow rate of 1.0 mL/min; inlet and interface temperature were 250 °C; and no split injection was used. The programmed heating conditions were as follows: the initial temperature was 40 °C, and the temperature rose to 150 °C at the rate of 4 °C/min. The temperature rose to 200 °C at the rate of 5 °C/min for 3 min, and the temperature rose to 230 °C at the rate of 20 °C/min for 5 min.

The conditions of mass spectrometry were as follows: electron ionization source, ionization voltage of 70 eV, ion source temperature of 250 °C, transmission line temperature of 250 °C, mass scanning range 30–400 *m*/*z*, and solvent delay time of 1 min.

Qualitative and quantitative analysis: Each peak in the total ion flow chromatogram and the mass spectrometry data of known substances in *NIST*, *Wiley*, and *Menalib* databases were retrieved and qualitatively identified on the basis that the degree of match was more than 800. The quantitative determination of the substance was replaced by the peak area and divided by the sample mass, shown in µg/kg sample.

### 2.8. Statistical Analysis

The data were analyzed via analysis of variance (ANOVA), with Duncan’s multiple range test using SPSS version 22.0 for Windows (SPSS Inc., Chicago, IL, USA). The results were expressed as the mean ± SE. The least-square means were separated by the least significant differences (*p* < 0.05).

## 3. Results

### 3.1. Variation in Oxidation Stability of the Longissimus Thoracis Muscles under Different Feeding Regimes

**Figure 1:**
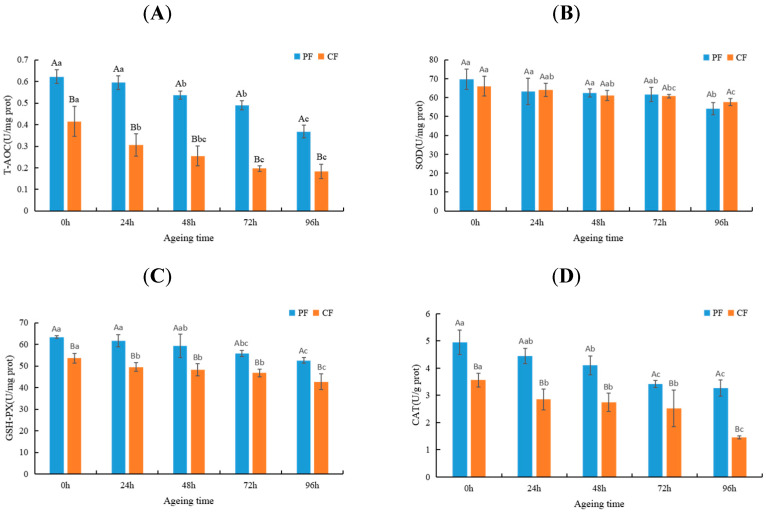
Changes in T-AOC (**A**), SOD activity (**B**), GSH-PX activity (**C**), and CAT activity (**D**) in LT muscle of the PF and CF during postmortem aging. The capital letters indicate significant differences in feeding regimes (*p* < 0.05), and the lowercase letters represent significant differences at different time points during postmortem aging (*p* < 0.05).

The level of antioxidant enzyme activity can be used as a biomarker of oxidative stress because it reflects the antioxidant state of the tissue [25]. To verify the effects of feeding regimes and aging on the antioxidant state of sheep meat, we evaluated the T-AOC (2A) and activities of the SOD (2B), GSH-PX (2C), and CAT (2D) in LT muscle (Figure 1). The T-AOC and activities of the GSH-PX and CAT in the PF were significantly higher than those in the CF (*p* < 0.05) because the fresh plants were rich in many antioxidants (selenium and vitamin E), which may increase the antioxidant activity in grazing sheep meat [26]. In addition, Rossetti et al. observed that exercise could improve the scavenging ability of free radicals in sheep muscle and induce the expression of higher antioxidant activity of antioxidant enzymes [27]. The antioxidant activity in both groups decreased significantly within 0–96 h after slaughter (*p* < 0.05). T-AOC, GSH-PX, and CAT in the CF decreased significantly within 0–24 h after slaughter, while those in the PF decreased significantly at 48 h after slaughter. This indicates that the oxidation stability changed later in the PF group than in the CF group during postmortem aging, which is consistent with the results of previous studies [28]. Collectively, the antioxidant activity of the PF was higher than that of the CF during postmortem aging, and the aging time was negatively correlated with the antioxidant capacity of sheep meat.

### 3.2. Variation in the Quality Attributes of Meat of during the Postmortem Aging of the Longissimus Thoracis Muscles under Different Feeding Regimes

Table 2 shows the effects of different regimes on meat quality attributes of Sunit sheep during postmortem aging, in which pH is an important index to evaluate meat quality during carcass maturation. The decline rate and degree of muscle pH affect muscle protein characteristics and then meat quality [29]. It can be seen from the table that the pH of the PF and CF decreased significantly at 0–24 h after slaughter, which was due to the decrease of pH due to the accumulation of lactic acid in the process of anaerobic glycolysis [30]. The pH24 of the CF was significantly higher than that of the PF (*p* < 0.05), which was similar to the results of [31]. This may be due to the higher activity of glycolytic enzymes in grazing sheep and the higher lactic acid produced after slaughter, which led to decrease in pH.

The color of meat is an important sensory feature that affects consumers’ purchase decisions in the market [32]. The L* is used to characterize the brightness of meat. The increase in brightness during muscle aging after slaughter is due to the weakening of protein structure and the increase of light scattering caused by protein degradation [33]. The L* of the CF was significantly higher than that of the PF at 0 h and 96 h after slaughter (*p* < 0.05). In the LT muscle of the CF, the L* values of 48 h, 72 h, and 96 h were significantly higher than those of 0 h, while those of the PF at 24–96 h were significantly higher than those of 0 h (*p* < 0.05), which indicated that the L* of sheep meat increased during postmortem aging, which was consistent with the results of Stanišić‘s research [34]. We found that in the process of postmortem aging, the feeding regimes had no significant effect on the a* of sheep meat (*p* > 0.05). Some studies have shown that the a* of muscle is affected by pH, which can affect the oxidation and reduction of myoglobin by adjusting the pH and can then change the meat color [35]. The reason why there is no significant difference in a* between the PF and CF may be that the change of pH under different feeding regimes is not enough to produce a significant difference. The a* at 24–96 h after postmortem was significantly higher than those at 0 h (*p* > 0.05), which was consistent with the results of Marino‘s research [33]. The possible reason is that postmortem aging can increase the permeability of myoglobin to oxymyoglobin and make muscle more easily obtain oxygen, thus becoming redder. In the LT muscle of the CF, the b * at 72 h after postmortem was significantly higher than that of the PF (*p* < 0.05). The reason for this may be the higher activity of antioxidant enzymes in the PF, which inhibited the degree of fat oxidation in the muscle after slaughter, lowering the b * and improving the stability of meat color [36]. The b* values of the CF and PF at 24–96 h after postmortem were significantly higher than those at 0 h (*p* < 0.05), which may be due to the oxidation of intramuscular fat in full contact with oxygen after death, thus increasing the b* [35].

Tenderness is not only an important attribute for consumers to be satisfied with the palatability of meat, but it is also the most important driver for consumers to purchase meat [37]. Shear force is used to evaluate the degree of meat tenderness during postmortem aging. With the extension of postmortem time, the shear force of the CF and PF began to increase and reached the maximum at 24 h. The possible reason is that calpain activity is low at low pH, which is not conducive for calpain to degrade muscle protein, and then, pH increases, calpain activity increases, and shear force decreases gradually [38]. The shear force of LT muscle in the PF was significantly lower than that in the CF at 0–72 h after slaughter. It may be that the oxidative stability of the PF was better than that of the CF after slaughter, which increased the activity of endogenous enzymes, thus promoting the rupture of myofibril and increasing the final meat tenderness [39]. At 96 h after slaughter, the difference of shear force between the PF and CF disappeared, which indicated that aging may eliminate the difference of meat tenderness caused by pre-slaughter factors.

Water retention is the key factor affecting the quality of sheep meat. The cooking loss rate of LT muscle in the CF was significantly higher than that in the PF (*p* < 0.05), which indicated that the PF had better water retention [40]. With the extension of postmortem aging time, the cooking loss rate of LT muscle increased significantly (*p* < 0.05), indicating that the water retention of meat decreased continuously during postmortem aging. This may be due to the fact that after the animals were slaughtered, the lactic acid produced by anaerobic glycolysis decreased the pH, which decreased to close to the isoelectric point of muscle protein, and the protein produced an electrostatic charge. The electrostatic repulsion between the molecules decreases so that the network is in a state of contraction, the internal space of the muscle becomes smaller, and the water retention is reduced [41].

Overall, the tenderness and water retention of the PF were better than those of the CF in the process of postmortem aging. Aging time has a great effect on the pH, tenderness, color, and water retention of sheep meat. After 24 h of aging, the pH and shear force of sheep meat began to increase, and the meat color also tended to be stable.

### 3.3. Variation in Fatty Acid Content during Postmortem Aging of the Longissimus Thoracis Muscles under Different Feeding Regimes

As shown in Table 3, total saturated fatty acids (SFAs) increased, and total monounsaturated fatty acids (MUFAs) and PUFAs decreased during postmortem aging in both groups of animals; Zhang reported similar results. The study hypothesized that the increase in SFAs may be due to the deterioration of long-chain PUFAs [42]. During postmortem aging, the reduction of MUFAs and PUFAs as flavor precursors could be due to the facilitation of oxidation. This finding is in accordance with the increase in the MDA value with the progress of postmortem aging. Yang concluded that the reduction of PUFAs during postmortem aging may contribute to the formation of volatile compounds [43].

In our study, the major fatty acids in sheep muscle were palmitic acid (C16:0), stearic acid (C18:0), oleic acid (C18:1), and linoleic acid (C18:2 n-6) in both groups. The main SFAs in meat are palmitic acid (C16:0) and stearic acid (C18:0), and their contents were not affected by the feeding regimes. This is consistent with Scerra et al. [44]. These fatty acids are essential for humans and are also essential prerequisites for the processing and storage of volatile compounds [45]. The oxidation of linoleic acid produces 2-pentylfuran, hexaldehyde, and 2-heptanone; the oxidation of oleic acid produces 1-octanol; and the oxidation of palmitic acid and oleic acid can produce 1-hexanol [46]. In the CF group, the most significant changes in the percentages of fatty acids, such as lauric acid (C12:0), myristic acid (C14:0), palmitic acid (C16:0), stearic acid (C18:0), oleic acid (C18:1), linoleic acid (C18:2 n-6), and arachidonic acid (C20:4 n-6), were observed from 0 to 24 h postmortem, indicating that the most dramatic variation in fatty acid composition occurred at 0–24 h; this may be due to the loss of dripping water, as these sheep were cut before the carcass was stiff.

The PF had a lower SFA content and higher contents of PUFAs and MUFAs than the CF (*p* < 0.05), indicating that the different feeding regimes had a significant effect on the fatty acid composition of sheep. This finding is similar to those of Wang, who suggested that the diversity of fatty acids in sheep raised on different feeding regimes might be associated with the differences in plants consumed [3]. Oleic acid is a heart-healthy fatty acid and is the main MUFA in the Western diet. Linoleic acid (C18:2 n-6) and alpha-linolenic acid (C18:3 n-3) are considered essential fatty acids. These fatty acids can only be obtained from the diet and cannot be synthesized by the body itself [47]. Through the body’s own functional metabolism, α-linolenic acid can be converted into eicosapentaenoic acid (EPA, C20:5 n-3) and docosahexaenoic acid (DHA, C22:6 n-3). However, their conversion efficiency is almost negligible, and therefore, dietary supplements are required [47]. In this study, the proportion of (C18:3 n-3) in the PF was higher than that in the CF, but it was not significant. The reason for this was probably that, although the (C18:3 n-3) concentration in pasture grass is higher than that of concentrated feed, the grazing period is shorter. During postmortem aging, the percentage content of DHA (C22:6 n-3) was substantially higher in the PF group, which also had much higher levels of linoleic acid (C18:2 n-6), eicosatrienoic acid (C20:3 n-6), and EPA (C20:5 n-3). These results are consistent with the results of the Kitessa [48], which may be related to the good antioxidant capacity of the PF.

Generally speaking, the proportion of unsaturated fatty acids in the PF was higher than that in the CF, and with the extension of aging time, the hydrolysis or oxidation of unsaturated fatty acids contributed to the deposition of volatile flavor compounds in sheep meat.

### 3.4. Variation in Lipid Peroxidation during Postmortem Aging of the Longissimus Thoracis Muscles under Different Feeding Regimes

**Figure 2:**
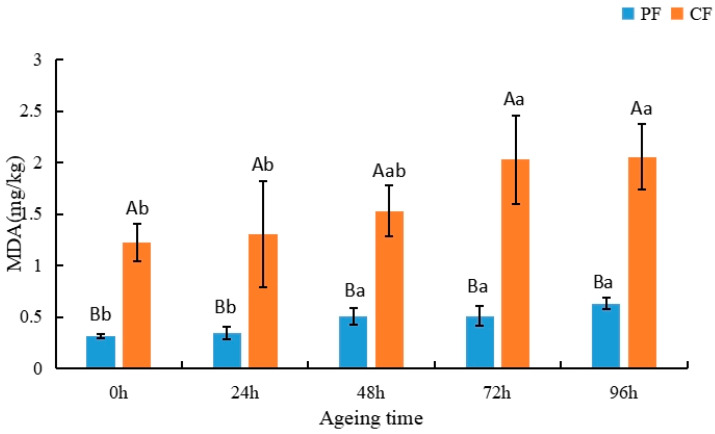
Changes in malondialdehyde content (mg/kg) in the LT muscle of the PF and CF during postmortem aging. The capital letters indicate significant differences in feeding regimes (*p* < 0.05), and the lowercase letters represent significant differences at different time points during postmortem aging (*p* < 0.05).

Malondialdehyde (MDA) is primarily derived from the decomposition of PUFAs [49]. Therefore, the degree of lipid peroxidation in products made from meat can be assessed according to MDA values [50]. In addition, lipid peroxidation is capable of producing certain volatile compounds [51,52]. Appropriate levels of lipid peroxidation can create desirable flavors, but over-oxidation can produce unfavorable flavors in the product and can even affect the quality and safety of the meat product [53]. As shown in Figure 2, lipid peroxidation (MDA values) in sheep significantly increased during the postmortem aging process. The progressive increase in MDA values indicates that lipid peroxidation progressively increased during postmortem aging. Initially, the MDA value of the CF was 1.23 mg/kg. After 96 h of postmortem aging, the value increased to 2.06 mg/kg. The results for the PF were similar, with the MDA value increasing from 0.32 to 0.63 mg/kg. MDA values in the CF were significantly higher than in the PF during postmortem aging (*p* < 0.05). Analogous findings were reported by Lou, who attributed the lower MDA values of the PF to the higher content of antioxidants in the grasses consumed during pasture feeding [8].

### 3.5. Variation in Volatile Organic Compounds during Postmortem Aging of the Longissimus Thoracis Muscles under Different Feeding Regimes

**Figure 3:**
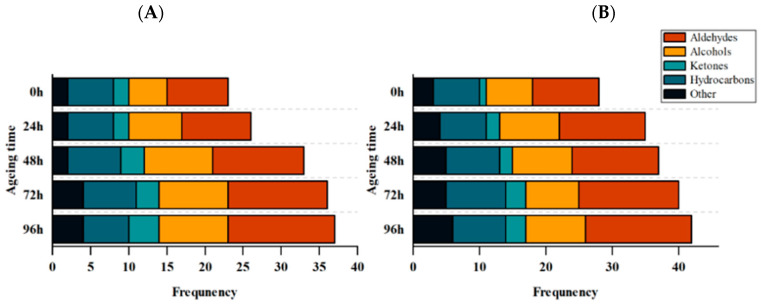
Changes in the species of volatile flavor substances derived from lipid oxidation in LT muscle of the PF and CF during postmortem aging. (**A**) PF and (**B**) CF. The capital letters indicate significant differences in feeding regimes (*p* < 0.05), and the lowercase letters represent significant differences at different time points during postmortem aging (*p* < 0.05).

Sheep meat is loved by many consumers because of its unique flavor. The flavor of mutton depends on the chemical composition of volatile (aroma) compounds. The type, quantity, and balance of flavor molecules are essential to the acceptability of meat flavor [54]. Volatile organic compounds, such as aldehydes, alcohols, ketones, phenols, etc., have been considered to be the cause of mutton flavor [55]. The biosynthesis of these volatile compounds involves a variety of pathways, including fatty acids, amino acids, terpenes, and carotenoids. Multiple biochemical pathways are responsible for releasing mixtures of volatile compounds from sheep meat [56]. For example, the oxidation of n-6 and n-9 PUFAs produces hexanal, heptanal, and nonanal [57]. Analysis of the contents and types of volatile organic compounds in lamb aged for different times under different feeding regimes were performed by gas chromatography-mass spectrometry (GC-MS) (Figure 3, Table 4). The results showed that aldehydes, alcohols, and ketones detected in this experiment were dominant in all volatile components. Using the *NIST* database, the volatile flavor compounds found in the PF at 0, 24, 48, 72, and 96 h after slaughter were 23, 26, 33, 36, and 37, respectively, and 28, 35, 37, 40, and 42 in the CF. The species of volatile organic flavor compounds in the PF were always lower than those in the CF. The types of volatile flavor compounds increased with the extension of aging time, which was consistent with the quantitative change trend of volatile flavor compounds during beef ripening determined by Watanabe et al. [58]. The possible reason for this trend is that during postmortem aging, the flavor precursor fatty acids in muscle are oxidized due to the decrease of antioxidant capacity, and some short-chain free fatty acids released will produce a strong odor. On the other hand, medium-chain and long-chain free fatty acids will be further oxidized to produce additional volatile flavor compounds, accelerating the formation of volatile flavor compounds during postmortem aging [42].

Table 4 shows the changes of total concentration of volatile compounds derived from lipid oxidation in sheep meat under different feeding regimes and postmortem aging time. Aldehydes are the most important volatile organic flavor components [59]. Most of it comes from oxidative hydrolysis of fat and very little from Maillard reaction of sugars [55]. The aldehyde contents originating from lipid oxidation in the PF and CF increased during postmortem aging. A previous study demonstrated that lower antioxidant enzyme activity led to a significant increase in volatile aldehyde content [60]. Alcoholic volatile organic compounds are mainly produced by the degradation of conjugated linoleic acid in muscle by lipoxygenase and hydroperoxide [61]. The total content of alcohols increased with the increase of aging time, and at the same time, the total content of alcohols in the CF was significantly lower than that in the PF at 0–48 h after slaughter (*p* < 0.05). Ketone volatile organic compounds are produced by lipid oxidation, hydrolysis, and microbial metabolism. Most of them have a creamy or fruit flavor, which has a positive effect on meat flavor [62]. The total content of ketones in the PF was significantly higher than that in the CF during postmortem aging (*p* < 0.05) and increased with the extension of aging time. Other fat oxidation products, including alkanes, acids, and furans, also showed an upward trend after slaughter.

To sum up, with the extension of aging time, the content of volatile flavor compounds caused by lipid oxidation in the PF and CF increased, which enriched the flavor of meat. The effect of feeding regimes on the total content of flavor substances was more significant at 0 h after slaughtering. The main factors leading to these differences were the differences in diet and exercise between the two groups, but with the extension of aging time, the difference in the content of flavor compounds between the two groups was basically eliminated.

## 4. Conclusions

The results showed that feeding regimes and postmortem aging time had significant effects on antioxidant activity, meat quality, fatty acid composition, lipid oxidation, and volatile compound formation of LT muscle in Sunit sheep. Sheep meat in the PF had better antioxidant activity, tenderness, water retention, and fatty acid composition. With the extension of aging time, the antioxidant capacity and the proportion of unsaturated fatty acids in sheep meat of the PF and CF gradually decreased, while the degree of lipid oxidation and the content of volatile flavor compounds increased gradually. At the same time, after 24 h of aging, the pH and shear force of sheep meat began to increase, and the color tended to be stable. The increase of lipid oxidation during aging leads to the increase of volatile compounds and promotes the formation of sheep meat flavor. In addition, aging can also eliminate the differences caused by feeding regimes to some extent. This study helps to further understand the effects of different feeding regimes and postmortem aging time on meat quality, antioxidant capacity, fatty acid composition, and volatile flavor components in Sunit sheep and provides theoretical reference and technical support for subsequent improvement of meat quality.

## Figures and Tables

**Table 1:** Chemical composition (g kg^−1^ dry matter) and fatty acid composition of the diets at different feeding regimes.

Item	PF	CF
Chemical composition		
Crude protein	139.8	193.8
Crude fat	30.0	47.0
Ash	42.8	79.1
Neutral detergent fiber	326.5	131.8
Fatty acid composition		
C14:0	2.7	5.5
C16:0	17.6	41.0
C16:1	0.4	1.1
C18:0	1.3	16.2
C18:1 cis-9	3.2	15.1
C18:2 n-6	12.9	14.8
C18:3 n-3	56.3	2.5

**Table 2:** Effects of different feeding regimes and postmortem aging times on the quality attributes in the LT muscles of sheep.

Item	Feeding Regimes	Ageing Time				
		0 h	24 h	48 h	72 h	96 h
pH	CF	6.04 ± 0.17 Aa	5.61 ± 0.03 Ab	5.65 ± 0.14 Ab	5.67 ± 0.04 Ab	5.69 ± 0.06 Ab
	PF	5.92 ± 0.27 Aa	5.52 ± 0.01 Bb	5.54 ± 0.09 Ab	5.57 ± 0.08 Ab	5.61 ± 0.08 Ab
	CF	68.74 ± 1.06 Ab	77.03 ± 1.14 Aa	63.24 ± 1.72 A	61.85 ± 1.62 A	59.84 ± 1.74 A
	PF	59.62 ± 1.43 Bb	69.76 ± 3.56 Ba	55.46 ± 1.61 B	54.71 ± 1.56 Bc	52.09 ± 1.46 c
L*	CF	34.42 ± 2.74 Ab	37.21 ± 2.43 Aab	38.19 ± 1.05 A	38.56 ± 2.34 a	40.03 ± 0.45 a
	PF	32.78 ± 1.15 Bb	36.49 ± 0.92 Aa	36.30 ± 0.12 A	36.23 ± 1.72 A	35.98 ± 0.68 B
a*	CF	19.22 ± 0.39 Ab	21.26 ± 0.53 Aa	20.82 ± 0.58 A	20.97 ± 0.59 A	21.05 ± 1.19 A
	PF	18.88 ± 0.34 Ab	21.10 ± 0.60 Aa	20.65 ± 0.61 A	20.47 ± 1.00 A	20.02 ± 0.50 A
b*	CF	3.66 ± 0.05 Ab	6.25 ± 0.50 Aa	6.93 ± 0.68 Aa	7.22 ± 0.61 Aa	7.34 ± 1.05 Aa
	PF	3.69 ± 0.48 Ab	6.19 ± 0.45 Aa	6.21 ± 0.59 Aa	6.16 ± 0.26 Ba	7.03 ± 1.10 Aa
Cooking loss (%)	CF	29.58 ± 2.61 Ac	33.07 ± 2.54 Ab	36.01 ± 0.49 A	37.27 ± 1.91 A	38.22 ± 1.12 a
	PF	21.69 ± 1.09 Bc	25.5 ± 1.09 Bb	31.77 ± 1.40 B	33.29 ± 1.64 Ba	34.25 ± 1.03 B

L*: Lightness; a*: redness; b*: yellowness. The capital letters indicate significant differences in feeding regimes (*p* < 0.05), and the lowercase letters represent significant differences at different time points during postmortem aging (*p* < 0.05).

**Table 3:** Effects of different feeding regimes and postmortem aging times on the fatty acid composition and levels in the LT muscles of sheep.

Fatty Acid (%)	Feeding Regimes	Ageing Time				
		2 h	24 h	48 h	72 h	96 h
C12:0	PF	0.63 ± 0.03 Ab	0.37 ± 0.07 Bd	0.44 ± 0.05 cd	0.53 ± 0.04 Abc	0.87 ± 0.05 Aa
	CF	0.49 ± 0.02 Bb	0.63 ± 0.01 Aa	0.48 ± 0.02 b	0.39 ± 0.01 Bc	0.47 ± 0.01 Bb
C14:0	PF	2.25 ± 0.02 Ac	2.06 ± 0.02 Ac	2.10 ± 0.22 Ac	2.77 ± 0.08 Ab	3.11 ± 0.05 Aa
	CF	1.61 ± 0.13 Ba	1.50 ± 0.03 Bb	1.28 ± 0.03 Bc	2.30 ± 0.02 Ba	2.26 ± 0.04 Ba
C14:1	PF	0.28 ± 0.17 Aa	0.35 ± 0.05 Aa	0.28 ± 0.05 Aa	0.22 ± 0.02 Ba	0.23 ± 0.01 Ba
	CF	0.24 ± 0.04 Ab	0.36 ± 0.02 Ab	0.37 ± 0.09 Ab	0.43 ± 0.02 Ab	0.42 ± 0.04 Ab
C16:0	PF	21.11 ± 1.54 Ab	21.93 ± 0.02 Ab	24.15 ± 0.01 Aa	23.81 ± 0.06 Aa	24.70 ± 0.22 Aa
	CF	21.34 ± 0.42 Ab	22.40 ± 0.37 Aa	22.94 ± 0.70 Aa	23.27 ± 0.12 Ba	23.09 ± 0.12 Ba
C16:1	PF	2.65 ± 0.39 Aa	2.40 ± 0.12 Aa	2.89 ± 0.13 Aa	2.69 ± 0.09 Aa	2.39 ± 0.22 Aa
	CF	2.29 ± 0.42 Aa	1.92 ± 0.03 Bab	1.15 ± 0.12 Bc	1.65 ± 0.15 Babc	1.40 ± 0.16 Bbc
C18:0	PF	11.02 ± 0.07 Bd	11.22 ± 0.19 Bd	12.50 ± 0.05 Bc	14.68 ± 0.02 Bb	15.34 ± 0.04 Ba
	CF	14.76 ± 0.08 Ad	16.50 ± 0.04 Ac	17.36 ± 0.76 Ab	18.39 ± 0.20 Ab	20.44 ± 0.19 Aa
C18:1	PF	45.66 ± 1.46 Aa	45.63 ± 0.12 Aa	43.91 ± 0.17 Bb	42.05 ± 0.69 Ac	41.58 ± 0.29 Ac
	CF	45.70 ± 0.42 Aa	44.37 ± 0.06 Bb	44.51 ± 0.06 Ab	42.26 ± 0.11 Ac	42.25 ± 0.31 Ac
C18:2(n-6)	PF	9.09 ± 0.58 Aa	8.73 ± 0.01 Aa	7.77 ± 0.03 Ab	7.55 ± 0.15 Ab	6.10 ± 0.15 Ad
	CF	7.77 ± 0.21 Ba	7.20 ± 0.16 Bb	6.97 ± 0.33 Ab	6.39 ± 0.09 Bc	4.95 ± 0.03 Bd
C20:0	PF	0.48 ± 0.01 Ab	0.46 ± 0.02 Ab	0.47 ± 0.06 Ab	0.48 ± 0.10 b	0.76 ± 0.04 Aa
	CF	0.29 ± 0.01 Bb	0.29 ± 0.01 Bb	0.15 ± 0.01 Bc	0.38 ± 0.03 a	0.37 ± 0.01 Ba
C18:3(n-6)	PF	0.49 ± 0.10 Ab	0.85 ± 0.03 Aa	0.79 ± 0.02 Ab	0.48 ± 0.02 Aa	0.55 ± 0.06 Ab
	CF	0.46 ± 0.01 Ab	0.26 ± 0.05 Bb	0.76 ± 0.19 Aa	0.17 ± 0.02 Bb	0.16 ± 0.01 Bb
C18:3(n-3)	PF	0.95 ± 0.05 Aa	0.76 ± 0.05 Ab	0.50 ± 0.07 Bc	0.73 ± 0.04 b	0.51 ± 0.03 Bc
	CF	0.86 ± 0.07 Aa	0.77 ± 0.01 Ba	0.96 ± 0.01 Aa	0.63 ± 0.06 b	0.65 ± 0.03 Ab
C20:3(n-6)	PF	0.65 ± 0.04 Aa	0.41 ± 0.01 Ac	0.24 ± 0.05 Ad	0.53 ± 0.02 Ab	0.59 ± 0.01 Ad
	CF	0.39 ± 0.10 Ba	0.33 ± 0.09 Ba	0.34 ± 0.18 Aa	0.56 ± 0.03 Aa	0.48 ± 0.01 Ba
C20:4(n-6)	PF	3.51 ± 0.14 Ab	3.78 ± 0.01 Aa	3.17 ± 0.08 Ac	2.71 ± 0.16 Ad	2.58 ± 0.09 Ad
	CF	3.06 ± 0.16 Aa	2.42 ± 0.43 Bbc	1.86 ± 0.07 Bc	2.53 ± 0.01 Ab	2.37 ± 0.12 Abc
C20:5(n-3)	PF	1.00 ± 0.04 Aa	0.80 ± 0.07 Ab	0.61 ± 0.02 Ac	0.58 ± 0.01 Ac	0.59 ± 0.02 Ac
	CF	0.58 ± 0.07 Bb	0.92 ± 0.04 Aa	0.73 ± 0.09 Aab	0.55 ± 0.15 Ab	0.62 ± 0.01 Ab
C22:6(n-3)	PF	0.24 ± 0.01 Aa	0.24 ± 0.02 Ac	0.19 ± 0.01 Ab	0.19 ± 0.01 Ab	0.13 ± 0.01 Ac
	CF	0.14 ± 0.01 Ba	0.12 ± 0.03 Ba	0.14 ± 0.01 Ba	0.08 ± 0.01 Bb	0.08 ± 0.01 Bb
Summary						
ƩSFA	PF	35.49 ± 0.66 Ba	36.04 ± 0.14 Ba	39.67 ± 0.27 Bb	42.27 ± 0.19 Bc	44.78 ± 0.33 Bd
	CF	38.50 ± 0.65 Ad	41.33 ± 0.35 Ac	42.20 ± 0.07 Ac	44.73 ± 0.31 Ab	46.63 ± 0.36 Aa
ƩMUFA	PF	48.59 ± 0.90 Aa	48.39 ± 0.05 Aa	47.07 ± 0.09 Ab	44.95 ± 0.58 Ac	44.39 ± 0.51 Ac
	CF	48.23 ± 0.27 Aa	46.64 ± 0.01 Bb	46.03 ± 0.15 Bb	44.34 ± 0.28 Ac	44.07 ± 0.50 Ac
ƩPUFA	PF	15.93 ± 0.77 Aa	15.57 ± 0.17 Aa	13.26 ± 0.15 Ab	12.78 ± 0.36 Ab	10.84 ± 0.06 Ac
	CF	13.27 ± 0.28 Ba	12.02 ± 0.37 Bb	11.77 ± 0.15 Bb	10.93 ± 0.05 Bc	9.30 ± 0.08 Bd

The capital letters indicate significant differences in feeding regimes (*p* < 0.05), and the lowercase letters represent significant differences at different time points during postmortem aging (*p* < 0.05).

**Table 4:** Effects of different feeding regimes and postmortem aging times on the concentration of volatile compounds derived from lipid oxidation in the LT muscles of sheep.

Furans (ng/g)	Feeding Regimes	Ageing Time				
		0 h	24 h	48 h	72 h	96 h
Aldehydes	PF	28.09 ± 0.81 Ac	33.53 ± 1.22 Ab	34.53 ± 1.22 Ab	38.53 ± 1.14 Aa	42.52 ± 3.08 Aa
	CF	23.43 ± 1.23 Bc	33.59 ± 2.06 Ab	35.59 ± 2.06 Ab	38.51 ± 1.15 Ab	43.59 ± 0.24 Aa
Alcohols	PF	35.74 ± 2.16 Bc	37.93 ± 1.14 Bc	43.24 ± 1.42 Bb	51.25 ± 3.14 Aa	49.84 ± 3.71 Aa
	CF	42.62 ± 1.45 Ac	47.36 ± 1.56 Ab	53.46 ± 2.63 Aa	52.81 ± 1.86 Aa	53.19 ± 2.44 Aa
Ketones	PF	6.03 ± 0.45 Ad	8.32 ± 0.34 Ac	10.19 ± 1.03 Ab	15.21 ± 2.31 Aa	16.42 ± 2.54 Aa
	CF	3.19 ± 0.18 Bc	6.23 ± 0.62 Bb	6.33 ± 0.32 Bb	8.49 ± 0.52 Ba	10.38 ± 1.15 Ba
Hydrocarbons	PF	2.05 ± 0.19 Ac	4.97 ± 0.29 Ab	7.82 ± 0.52 Aa	5.21 ± 0.52 Ab	7.32 ± 0.31 Aa
	CF	2.02 ± 0.10 Bd	5.27 ± 0.09 Ab	5.65 ± 0.32 Bb	4.12 ± 0.60 Ab	8.83 ± 0.34 Aa
Acids	PF	2.46 ± 0.72 Bc	4.02 ± 1.65 Ab	5.69 ± 1.20 Ab	8.12 ± 1.54 Aa	8.43 ± 2.11 Aa
	CF	3.62 ± 1.79 Ad	5.26 ± 1.49 Ac	6.12 ± 1.26 Ac	8.44 ± 0.54 Ab	10.47 ± 1.13 Aa
Furans	PF	1.56 ± 0.12 Ad	2.93 ± 0.11 Ac	5.69 ± 1.20 Aa	3.38 ± 1.55 Ab	5.22 ± 0.41 Aa
	CF	1.01 ± 0.12 Bc	3.18 ± 0.11 Ab	2.97 ± 0.11 Bb	3.46 ± 0.72 Ab	5.15 ± 1.53 Aa

The capital letters indicate significant differences in feeding regimes (*p* < 0.05), and the lowercase letters represent significant differences at different time points during postmortem aging (*p* < 0.05).

## Data Availability

Not applicable.
